# Three-year follow-up of a randomised controlled trial to reduce excessive weight gain in the first two years of life: protocol for the POI follow-up study

**DOI:** 10.1186/s12889-016-3383-4

**Published:** 2016-08-11

**Authors:** Rachael W. Taylor, Anne-Louise M. Heath, Barbara C. Galland, Sonya L. Cameron, Julie A. Lawrence, Andrew R. Gray, Gerald W. Tannock, Blair Lawley, Dione Healey, Rachel M. Sayers, Maha Hanna, Kim Meredith-Jones, Burt Hatch, Barry J. Taylor

**Affiliations:** 1 Department of Medicine, University of Otago, PO Box 56, Dunedin, 9054 New Zealand; 2 Department of Human Nutrition, University of Otago, Dunedin, New Zealand; 3 Department of Women’s and Children’s Health, University of Otago, Dunedin, New Zealand; 4 Department of Preventive and Social Medicine, University of Otago, Dunedin, New Zealand; 5 Department of Microbiology and Immunology, University of Otago, Dunedin, New Zealand; 6 Department of Psychology, University of Otago, Dunedin, New Zealand

**Keywords:** Infant, Child, Obesity, Prevention, Sleep, Diet, Physical activity, Self-regulation

## Abstract

**Background:**

The Prevention of Overweight in Infancy (POI) study was a four-arm randomised controlled trial (RCT) in 802 families which assessed whether additional education and support on sleep (Sleep group); food, physical activity and breastfeeding (FAB group); or both (Combination group), reduced excessive weight gain from birth to 2 years of age, compared to usual care (Control group). The study had high uptake at recruitment (58 %) and retention at 2 years (86 %). Although the FAB intervention produced no significant effect on BMI or weight status at 2 years, the odds of obesity were halved in those who received the sleep intervention, despite no apparent effect on sleep duration. We speculate that enhanced self-regulatory behaviours may exist in the Sleep group. Self-regulation was not measured in our initial intervention, but extensive measures have been included in this follow-up study. Thus, the overall aim of the POI follow-up is to determine the extent to which augmented parental support and education on infant sleep, feeding, diet, and physical activity in the first 2 years of life reduces BMI at 3.5 and 5 years of age, and to determine the role of self-regulation in any such relationship.

**Methods/design:**

We will contact all 802 families and seek renewed consent to participate in the follow-up study. The families have received no POI intervention since the RCT finished at 2 years of age. Follow-up data collection will occur when the children are aged 3.5 and 5 years (i.e. up to 3 years post-intervention). Outcomes of interest include child anthropometry, body composition (DXA scan), diet (validated food frequency questionnaire), physical activity (accelerometry), sleep (questionnaire and accelerometry), and self-regulation (questionnaires and neuropsychological assessment).

**Discussion:**

Our follow-up study has been designed primarily to enable us to determine whether the intriguing benefit of the sleep intervention suggested at 2 years of age remains as children approach school age. However, cohort analyses will also investigate how BMI, self-regulation, and sleep consolidation develop during the early years. This information will be valuable to researchers and policy makers progressing the field of early childhood obesity prevention.

**Trial registration:**

ClinicalTrials.gov number NCT00892983.

## Background

Globally, it is estimated that 42 million children are overweight or obese, illustrating the seriousness of this public health challenge [1]. The prevalence of childhood obesity has increased substantially since the 1970s and there is little evidence to suggest these rates are tapering off [2, 3]. It is clear that New Zealand (NZ) children are following this global trend, with 9.5 % of children being obese by the time they are only 2–4 years of age and a further 20 % being overweight [4]. In addition to the immediate adverse effects of obesity in infancy and toddlerhood [5], it is also a strong risk factor for overweight later in life, and the associated health effects [6]. Determining the most effective tools for reducing excessive weight gain early in life is paramount if we are to prevent obesity before it becomes entrenched [7].

The early years are a time of immense change when fundamental behaviours including those around eating, sleeping, and physical activity are established. It is apparent that overweight and obesity develop from the complex interaction of multiple factors [8, 9], including biological effects (e.g. genetic susceptibility [10], and an ‘obesogenic’ gut microbiome [11]), behavioural factors (e.g. dietary, physical activity, sedentary and sleep habits [12, 13]), as well as cultural, social, environmental and economic influences [14, 15]. Evidence also suggests that parenting and discipline practices [16–18], mental health [19], and infant temperament [20, 21] may influence the development of obesity in young children. It has recently become apparent that many of these factors are influenced by self-regulation – the ability to control one’s behaviour without external assistance [22, 23]. To gain traction in reducing childhood obesity rates, interventions need to modify a wide range of these factors.

Relatively few obesity prevention trials have been undertaken in the first 2 years of life [24, 25], and many have been small [26, 27] or used non-randomised designs [28, 29]. However, three large randomised controlled trials (RCTs) have recently been undertaken in Australia. All three have targeted nutrition and eating behaviours in families [30–32] with two also addressing physical activity and sedentary behaviour [30, 31]. However, only one of these studies saw benefits in terms of relative body weight at the end of the trial [30]. This home-based intervention in mothers from disadvantaged areas of Sydney reported body mass index (BMI) values to be 0.29 kg/m^2^ (95 % confidence interval −0.55 to −0.02; *P* = 0.04) lower in intervention compared with control children at 2 years of age, with differences observed in several behavioural variables that might explain this effect on weight status [30]. Disappointingly, this intervention benefit was only transient, with significant effects no longer apparent when the children were 5 years of age [33]. Neither of the remaining studies observed a significant intervention effect on BMI at 18 [31] or 24 [32] months of age, nor at follow-up at 5 years of age [34]. Interestingly, all three studies had little impact on nutritional intake or levels of physical activity in children, although time spent watching television appeared to be modifiable [30, 31, 35]. Although it is not clear why it might be so difficult to influence nutrition or activity levels early in life, it is feasible that other priorities such as infant crying [36] and sleep problems [37] are more important to parents at this age.

In 2009, we commenced the POI (Prevention of Overweight in Infancy) RCT in 802 Dunedin families designed to reduce the number of children with excessive weight gain in the first 2 years of life [38]. Participants were randomised to one of four groups: Control (usual care), FAB (food, physical activity and breastfeeding), Sleep, or Combination (received FAB and Sleep interventions). All four groups received standard Maternity and Well Child care [39], and the three intervention groups received additional guidance and support on the relevant behaviours (breastfeeding, diet, physical activity, sleep) as detailed previously [38, 40, 41]. The POI study, and the three large controlled trials from Australia (Healthy Beginnings [42], Nourish [43], and InFANT [44]), are combining their data to form a prospective meta-analysis of the feasibility and effectiveness of early obesity prevention efforts (Early Prevention of Obesity in Children, EPOCH) [45]. Care has been taken to ensure that the study design, participants, intervention, comparators and outcome measures are similar across all four studies.

All four EPOCH studies aimed to modify BMI in the first 2 years of life through food, parenting and physical activity interventions. However, in addition to targeting these behaviours, the POI study also included a sleep intervention arm. Whether sleep interventions can affect growth trajectories is not known, but is an area of great interest internationally [46]. We know that adequate sleep in infancy and childhood is important for behaviour, learning and family functioning [47] but a wealth of data now link poor sleep habits with an increased risk of overweight in childhood as well [13, 48–50]. Despite this, we know relatively little about whether it is possible to change sleep behaviour and ultimately affect growth because very few sleep interventions have been published to date [26, 51–53]. Our brief POI intervention (2 core visits) focused on developing appropriate sleep habits from birth, including the ability of the infant to regulate their own sleep, i.e. to fall asleep when tired without external aids such as being fed to sleep or being rocked in a parent’s arms [54]. At 2 years of age, we observed no significant intervention effect on mean BMI across the four study groups. However, an exploratory analysis demonstrated that when the two groups receiving the sleep intervention (Sleep and Combination) were combined, the sleep intervention halved the odds of obesity (BMI ≥95th percentile) compared to the groups who did not receive the sleep intervention (Control and FAB) [55]. It thus seems opportune to follow-up this cohort to determine, first, whether this effect on obesity is sustained, and second, the mechanism by which a sleep intervention might beneficially affect weight in young children.

While it is clear that sleep is related to body weight in children [13, 48–50] the mechanisms are poorly understood [56]. The development of self-regulation, which mainly occurs in the first 4 years of life, seems fundamental in setting an individual’s life trajectory [57] and it appears that some of the earliest demonstrations of self-regulation are the ability to go to sleep without external assistance, and to match energy intake with energy requirements. Infants who are able to regulate their own sleep have healthier sleep-wake patterns later in life [58], and general measures of self-regulation have also been associated with healthier body weights in children [59–61]. How infants regulate their own sleep is difficult to measure by questionnaire, and objective measurements using video-cameras are not feasible in large trials [62]. However, we have included well-validated measures of general and sleep self-regulation in the follow-up study proposed here. Child measures of inhibitory control and language (core skills associated with self-regulation) will be assessed using the Developmental Neuropsychological Assessment (NEPSY) subsets of statue, comprehension, phonological processing, and word generation [63], and the Head-Toes-Knees-Shoulders task [64], a measure of behavioural regulation which also assesses inhibition. These findings will be augmented with age-appropriate measures of the self-regulation of sleep (self-settling to sleep, awakenings from sleep, consolidated sleep duration), and energy balance (dietary intake, parental beliefs about the child’s ability to regulate their own energy intake, and physical activity assessed using accelerometry).

Other factors of interest in child growth include physical activity, diet and the gut microbiota. How physical activity patterns develop is of interest given the low rates of physical activity seen in some [65, 66], although not all [67], studies in young children, and the observation that marked declines in activity are apparent even in the preschool years [68]. Accelerometers are increasingly being used to measure physical activity in children under five years of age [69], although rarely in those less than 3 years of age [70, 71]. 24-h accelerometry data (over 5 days) were collected at 6, 12 and 24 months as part of POI to measure both sleep and physical activity [72], and extending these measures in our follow-up study will allow us to examine multiple questions of interest regarding the early development of physical activity and inactivity and sleep. How the gut microbiota develop in early life and the potential relationship with obesity is of intense interest worldwide [73]. Despite clear differences in bacterial composition between lean and obese mice, corresponding findings in studies of humans have been considerably more mixed [74–76]. However, several longitudinal studies have now demonstrated that variation in gut microbiota during infancy is related to overweight status at older ages [77–79], although existing studies contain few subjects [77, 78] or use parent-reported BMI values [79] which have known inaccuracies [80]. Many studies are also unable to account for dietary intake, which clearly alters the bacterial composition of the microbiome [76]. Although it remains to be seen whether variation in gut microbiota is a cause or consequence of obesity [81], our prospective study includes a large number of children whose diet and lifestyle since birth has been well documented.

The overall aim of the POI follow-up is to determine the extent to which augmented parental support and education on infant sleep, feeding, diet, and physical activity in the first 2 years of life reduces BMI at 3.5 and 5 years of age, and to determine the role of self-regulation in any such relationship.

The key objectives are to determine whether the POI ‘Sleep intervention’ or ‘FAB intervention’ from late pregnancy to 2 years of age result in differences in:

- BMI z-score at 3.5 and 5 years of age (primary objective)
- Self-regulation (overall, sleep-related, or energy) at 3.5 and 5 years of age
- Other weight indices (BMI, prevalence of overweight or obesity, waist circumference) at 3.5 and 5 years of age
- Body composition at 5 years of age
- Sleep (timing, duration, awakenings, problems) at 3.5 and 5 years of age
- Physical activity at 3.5 and 5 years of age
- Diet at 3.5 and 5 years of age.

Additional objectives include determining, in a cohort analysis, possible predictors of BMI, self-regulation (overall, sleep-related, energy), and sleep consolidation (timing, duration, awakenings, problems) at 3.5 and 5 years of age.

## Methods/Design

### Study design and participants

The POI study was a four-arm RCT controlled trial assessing whether additional education and support on sleep (Sleep group); breastfeeding, diet, and physical activity (FAB group); or both (Combination group) reduced excessive weight gain from birth to 2 years of age compared to usual care (Control group) [38]. All mothers who had booked into the single maternity hospital (>97 % of all births) serving the city of Dunedin, NZ, between May 2009 and November 2010 were invited to participate in the POI study when they were 28–30 weeks gestation. Exclusion criteria applied before birth were: home address outside Dunedin, planning to move from Dunedin in the next 2 years, booked into the maternity centre after 34 weeks gestation, or unable to communicate in English or Te Reo Māori (language of the indigenous (Māori) ethnic group of NZ). Exclusion criteria applied after birth were: identification of a congenital abnormality likely to affect feeding or growth, or birth before 36.5 weeks gestation. All participants enrolled in the original POI intervention will be invited to participate in follow-up measurements by research staff. The study was approved by the University of Otago Human Ethics Committee (12/274) and written informed consent will be obtained from participants before commencing the first follow-up visit at 3.5 years of age.

### Sample size

Retention at 2 years was 86 % (*n* = 686/802) which is higher than was anticipated in our original power calculations (20 % loss to follow-up was assumed) [38]. Based on this, we anticipate retaining at least 75 % of the original cohort through to age five, giving us an estimated sample size at 5 years of at least 600 participants. This would provide 80 % power to detect a difference in BMI of 0.3 kg/m^2^ or larger at 5 years of age between any pair of the four groups using a two-sided test at the 0.05 level, based on earlier data from this research group suggesting a standard deviation of 0.8 [49]. This sample size would also allow 80 % power for detecting differences in self-regulation scores of 0.35 standard deviations between any two arms of the trial (falling between a “small” and “moderate” effect size) and detecting correlations at the study level of 0.12 or larger between continuous measures (e.g. between parenting style at 12 months and self-regulation at 5 years and between consolidated sleep hours at 6 months, and self-regulation at 3.5 and 5 years) using two-sided tests at the 0.05 level.

### Data collection

All data will be collected via two clinic visits lasting 60–90 min, one when the child is 3.5 years, and the other when they are 5 years of age. In brief, the child and parent(s) will have their height, weight and waist circumference measured, and the parent completes the questionnaire while the child undergoes the neuropsychological measures of self-regulation and a hair sample is taken (see below). One week later, parents will be contacted and the accelerometer, remaining questionnaire items, 24-h microbiota food frequency questionnaire, and stool sample will be collected. The dual-energy x-ray absorptiometry (DXA) scan will occur at a separate visit within two weeks of the clinic visit (5 years of age only). Parents will be compensated with a $20 grocery voucher for attending the clinic visit and a $10 grocery for completing the take-home questionnaire. Children will be offered a story book of their choice as a gift. Families who have since left the area but want to continue participating will be able to arrange to have their child (and themselves) weighed and measured at a medical centre, and complete online questionnaires. No other measurements will be obtained for these out-of-town participants.

### Outcome measures

The timing of the outcome measures is presented in Tables 1 and 2. All measures are undertaken by trained research staff blinded to original group allocation.

**Table 1:** Outcome measures used to determine the effects of the POI intervention in children at 3.5 years and 5 years of age

				Age	
	Measure	Method	Objectives	3.5 years	5 years
Anthropometry	Height, weight	WHO Growth standards [83]	a	Child	Child
	BMI	Calculated	a	Child	Child
	BMI-for-age z-score	WHO Growth standards [83]	c	Child	Child
	Prevalence of overweight	BMI z-score ≥85th but <95th percentile	c	Child	Child
	Prevalence of obesity	BMI z-score ≥ 95th percentile	c	Child	Child
	Waist circumference	WHO Growth standards [83]	c	Child	Child
Self-regulation	Overall self-regulation	NEuroPSYchological Assessment (NEPSY) [63]	b	Child	Child
		Head-Toes-Shoulders-Knees task [64]	b	Child	Child
		Behavior Assessment System for Children (BASC) [85]	b	Child	Child
		Child Behavior Questionnaire [86]	b	Child	Child
	Sleep-related self-regulation	Researcher generated	b	Child	Child
	Energy self-regulation	Children’s Self-Regulation in Eating scale [87]	b	Child	Child
Body composition	Body fat %	Dual-energy X-ray absorptiometry (DXA) scan	d	-	Child
	Lean mass %	Dual-energy X-ray absorptiometry (DXA) scan	d	-	Child
Sleep	Sleep timing	Accelerometry [104]	e	Child	Child
	Sleep duration	Accelerometry [104]	e	Child	Child
	Awakenings	Accelerometry [104]	e	Child	Child
	Sleep problems	Researcher generated	e	Child	Child
	Sleep hygiene	Researcher generated	e	Child	Child
	Sleep disturbances	Sleep Disturbance Scale for Children [94]	e	-	Child
Physical activity	Physical activity	Accelerometry [72]	f	Child	Child
	Sedentary behaviour	Researcher generated	f	Child	Child
Diet	Dietary pattern scores	Food frequency questionnaire [96]	g	Child	Child
	Nutrient intake	Food frequency questionnaire [95]	g	-	Child

**Table 2:** Additional measures collected to determine the predictors of BMI, self-regulation, and sleep consolidation at 3.5 years and 5 years of age

			Child		Parent	
	Measure	Method	3.5 years	5 years	Child age 3.5 years	Child age 5 years
Child gut microbiota	Gut microbiota	From stool sample	Child	Child	-	-
Child mental health	Child depression	BASC-2 [85]	Child	Child	-	-
	Child anxiety	BASC-2 [85]	Child	Child	-	-
	Child stress	Hair cortisol [99]	-	Child	-	-
Parenting practices	Discipline	Researcher generated	-	-	Mother	Mother
Maternal mental health	Maternal depression	Depression, Anxiety and Stress Scale (DASS 21) [100]	-	-	Mother	-
	Maternal anxiety	Depression, Anxiety and Stress Scale (DASS 21) [100]	-	-	Mother	-
	Maternal stress	Depression, Anxiety and Stress Scale (DASS 21) [100]	-	-	Mother	-

#### Anthropometry (objectives a and c)

Trained measurers will conduct all anthropometric measurements following standard protocols [82]. Weight will be obtained with children wearing standard clothing (singlet and underwear) using regularly calibrated electronic scales (Tanita WB-100 MA/WB −110 MA). Height will be measured using a Harpenden stadiometer (Holtain Ltd, UK). Waist circumference will be measured at the minimum circumference between the rib cage and the iliac crest using a Rosscraft Anthropometric tape (Rosscraft Innovations Inc, USA). A third measurement will be made if duplicate measures are not within 0.1 kg for weight, 0.7 cm for height, and 1.0 cm for waist circumference (with the mean of the two closest measurements used in such cases) [38]. Weight-for-age z-score and BMI-for-age z-scores will be calculated using the WHO growth standards [83], with overweight defined as a BMI-for-age z-score ≥85th to <95th percentile and obesity defined as ≥95th percentile.

#### Self-regulation (objective b)

*Overall self-regulation* will be determined using the laboratory-based measures NeuroPSYchological Asssessment (NEPSY-2) [63] and the Head-Toes-Knees-Shoulders task [64]. The NEPSY-2 is a test battery assessing numerous areas of neuropsychological functioning. It is well-normed, reliable, and appropriate for use with 3–5 year-old children. Five subtests from this battery will be used in this study to assess targeted areas of neuropsychological functioning: ‘Statue’ which measures inhibitory control, ‘Comprehension of Instructions’ which assesses working memory, ‘Visuomotor Precision’ which measures inhibition and fine motor control, and ‘Phonological Processing’ and ‘Word Generation’ which both assess language development. In addition, children will complete the Head-Toes-Knees-Shoulders task, a measure of behavioural regulation and inhibition [64, 84]. As well as these laboratory measures, self-regulation will also be assessed using parent-report questionnaires to assess children’s day-to-day abilities to self-regulate behaviour outside the setting of the study assessment session. These questionnaires include the Behavioral Assessment System for children (BASC-2) [85] and the Children’s Behavior Questionnaire (CBQ) [86]. The BASC-2 is a well-validated and normed scale designed to assess wide-ranging areas of child functioning, as rated by parents and/or teachers. At both ages four subsets of the BASC will be assessed, namely ‘Hyperactivity’, ‘Attention Problems’, ‘Emotional Self-Control’, and ‘Executive Functioning’. The CBQ is an extensively used and validated measure of child temperament and items load into 3 broad factors: ‘Surgency’ which is a measure of positive affect and approach, ‘Negative Affect’ which indicates sensitivity to frustration and punishment, and ‘Effortful Control’ which evaluates attentional self-regulation. The latter two factors will be used as indicators of self-regulatory abilities.

*Sleep-related self-regulation* will be assessed using brief questions asking parents how often (six-point likert scale) their child falls asleep on their own, requires a parent to hold them or be in the room in order to fall asleep, has difficulty falling asleep, and stays in their own bed all night.

*Energy self-regulation* will be assessed using a brief eight-item questionnaire [87], which asks parents to agree or disagree (five-point likert scale) with eight statements covering issues such as the child knowing when they are full, eating when they are already full, and not eating snacks if they are already full.

#### Body composition (objective d)

Body composition will be assessed by dual-energy x-ray absorptiometry (DXA) at 5 years of age only. All DXA measurements will be performed and analysed by one experienced operator with a Lunar Prodigy scanner (software package 16.0; Lunar, Madison, WI) using standard procedures. The scanner determines total fat mass (kg) and the fat content (kg) of specific anatomical regions including trunk and extremity fat (automatic default regions) and central and peripheral fat (manual regions of interest) [88, 89]. In our laboratory the CVs for repeated in vivo scans on ten adults were 1.8 % for fat mass, 1.8 % for fat percentage, and <2.5 % for all regional measurements. The scans are well tolerated by children at this age (>95 % usable scans).

#### Sleep, physical activity and sedentary behaviour (objectives e and f)

Measures of habitual physical activity (counts per minute, time spent at different intensities of activity) and 24 h-sleep patterns (sleep-wake timing, sleep duration, night awakenings, overnight sleep efficiency, daytime naps) will be measured by five consecutive days of accelerometer recording including at least one weekend day (Acticals, Mini-Mitter, Bend, OR) [49] in all children. The accelerometer will be fitted to the child during the clinic visit. Children will be asked to wear the accelerometer 24 h a day for the full 5 days. The accelerometers are initialised using 15 s epochs and a valid day is defined as at least 8 h of wear time. Data will be cleaned and scored using an automated script developed in MATLAB (MathWorks, Natick, MA, USA). The program is initiated using a “time flag” for sleep onset and for sleep offset, approximately half an hour before the average bedtime and wake times for this age group. To detect sleep and wake states the count-scaled algorithm [90] is activated to detect wake “events” as the last of 15 continuous minutes of sleep followed by 5 min of awake and sleep “events” as the start of 15 continuous minutes of sleep preceded by 5 min of awake. Nap screening rules will be applied to the data for the 3.5 year olds to identify naps as sleep periods that occurred during the period of 9 am to 5 pm, where a sleep period is defined as at least 20 min of sleep, preceded by 5 min of awake and using a minimum sleep time threshold of 30 min [91]. Non-wear time applies to the period of time between sleep offset (morning wake) and bedtime and is defined as 20 min of consecutive zeros. Standard sleep-wake variables including nap counts and timing will be calculated using the automated script within MATLAB. Subjective measures of sleep will be collected by questionnaire. Data will be analysed for physical activity during waking hours only and expressed as both counts/min (measured in 15 s epochs) and in terms of intensity of activity (sedentary, light, moderate, vigorous) using the cutoffs of Evenson et al. [92] as recently recommended [93].

Data on parents’ perceptions of their child’s sleep as problematic, sleep hygiene covering the pre-sleep routine (for example bathing, reading, quiet time, use of electronic media), the sleep environment (who the child sleeps with, bedding and lighting arrangements), and sleep disturbances (snoring, restless legs, sleepwalking, nightmares and night terrors) will be collected by questionnaire. Parents will also complete the Sleep Disturbance Scale for Children when their child is 5 years of age [94]. Information on children’s electronic media use will also be collected from all children via questionnaire. This will include: the variety of media in the house (television, DVD, computer, tablet, cellphone, ‘iPod’), usual time spent on each device by the child, family rules around viewing, and viewing situations such as who the child was with, and where the device was viewed [38].

#### Diet (objective g)

In order to minimise respondent burden and thus achieve higher rates of participant retention, dietary intake (food pattern scores and nutrient intake) will be assessed using an interviewer administered food frequency questionnaire (FFQ) rather than diet records or 24-h recalls. At both 3.5 and 5 years of age, the FFQ will be based on the EAT FFQ that has been validated for measuring both nutrient intake [95] and dietary patterns [96] in young children. At 3.5 years, the FFQ will collect data on the frequency (13 frequency categories: ‘not eaten this month’ to ‘five or more times a day’) with which 90 food items have been consumed over the past month. At 5 years of age, the EAT5 FFQ will collect data on amount eaten as well as frequency of intake over the past month (ten frequency categories: ‘not eaten this month’ to number of times a day). Amounts will be collected as number of specified units (e.g. 1 biscuit), or volume (e.g. ml of rice) with the parent using dried beans or rice to demonstrate a typical portion size for that food. The 113 foods in the EAT5 FFQ will include foods from the original EAT FFQ that are still relevant at 5 years of age (i.e. excluding infant foods) with additional foods that are rich sources of food components that may influence the gut microbiota (i.e. resistant starch, polyphenols, choline). In addition, at 5 years of age the parent will be asked to complete a self-administered recall when they collect their child’s faecal sample (see next section). This recall will refer to the day before the sample was collected and is the same as the EAT5 FFQ but asks for frequency of intake only, not amount eaten.

#### Additional measures

##### Gut microbiota

A small stool sample (approximately 5 g) will be collected from each participant at 3.5 and 5 years. The sample will be placed into a specially provided freezer flask and stored in the home freezer before retrieval by POI research staff following notification of sample collection. The sample will be stored at −80 °C for later analysis of the composition of the stool microbiota. These analyses will focus on bacterial community phylogenetics using high throughput sequencing of amplicons generated from the 16S rRNA gene. DNA will be extracted from stool using the MoBio PowerSoil DNA Isolation kit according to the Human Microbiome Project standard operating procedures (http://www.hmpdacc.org/tools_protocols/tools_protocols.php). Genomic DNA will be submitted to Argonne National Laboratories for barcoded amplification of the V4 region of the bacterial 16S rRNA gene and sequencing on a MiSeq (Illumina) instrument. DNA sequences will be processed using the QIIME v. 1.9.1 suite of programs [97]. Genus level taxonomy will be obtained by filtering Operational Taxonomic Unit (OTU) tables containing taxonomic data generated using the RDP classifier, at a genus level, extracting representative sequences and using BLAST to identify genus level matches within the NCBI database. Alpha- and beta-diversity analysis of phylogenetic data will compare the coverage and richness (number of phylotypes), and similarity or difference in the composition of the stool microbiota between subject groups. Finally, the proportions of the major bacterial families and genera comprising the stool microbiota will be compared between normal and overweight children.

##### Child mental health

The child depression and anxiety subscales of the BASC-2 questionnaire will be used to assess child mental health. At the 5 year clinic appointment, a small sample of the child’s hair will be collected for measurement of hair cortisol as an indicator of chronic stress. Hair samples (around 100 hairs in total) will be obtained from the vertex posterior region (the back of the head in a line between tops of ears) of the scalp, and stored for later analysis of cortisol [98, 99].

##### Parenting practices

Discipline will be assessed using one question: “please state which of the following you have done to get your child to do, or to stop doing, something over the past week?” followed by a list of 14 common discipline strategies on which respondents will indicate the number of days (0 to 7) that strategy had been used in the last week.

##### Maternal mental health

Maternal mental health will be determined using the Depression, Anxiety and Stress Scale (DASS 21), a widely used 21 item quantitative measure covering depression, anxiety (symptoms of psychological arousal), and stress (the more cognitive, subjective symptoms of anxiety) [100].

Four additional measures will be collected but are not discussed here as they will not be part of the main analyses: child salivary oxytocin (5 years of age), maternal salivary oxytocin (child 3.5 years of age), child blood pressure (3.5 and 5 years of age), and awake heart rate variability in children lying supine followed by heart rate responses to a 70° head-up tilt test (3.5 and 5 years of age).

### Analysis

For the key objectives, data will be analysed using linear, logistic (binary or ordinal), and Poisson (or negative binomial) regression models to compare the four arms of the trial in terms of continuous, categorical (binary or ordinal), and count outcomes respectively, with a random effect for participant along with terms for age and a group-by-age interaction added when longitudinal outcomes are analysed. As analyses at 2 years did not find consistent evidence in support of interactions between the Sleep and FAB interventions, we also plan to investigate outcomes using combined Sleep and combined FAB groups. A two stage process will be used, first investigating whether there is evidence for such an interaction (a reparameterisation of the four group model) and if not, the Sleep and FAB intervention terms will be investigated without the interaction included, although this process will increase the Type I error rates beyond the nominal level [101]. All *P*-values will be reported using three decimal places and *p*-values less than 0.001 will be reported as ‘*p* < 0.001’. Two-sided *p* < 0.05 will be considered to indicate statistical significance and no adjustments for the multiple outcomes are planned. Post-hoc comparisons between the three intervention and one control groups will only be performed where the overall (Wald) test is statistically significant. Where available, baseline values will be included in models and all models will include the stratification variables [102]. Standard model diagnostics will be used and log-transformations used where this improves residual normality and/or homoscedasticity for continuous outcomes. Homser-Lemeshow tests will be used for binary logistic regression models and proportionality will be assessed in ordinal logistic regression models using generalised ordinal logistic regression. Overdispersion will be examined using a likelihood ratio test for count outcomes, with negative binomial regression used when this is significant. Where model residuals remain unacceptable for continuous outcomes, quantile regression, including mixed quantile regression where appropriate, will be used instead to model medians.

Similar models will be used for the additional objectives with the study group included but not of direct interest in itself. No subgroup analyses are currently planned. All effect sizes will be accompanied by 95 % confidence intervals and these will be used to assess clinical significance alongside determination of statistical significance. As missing data is expected to be around a quarter of the original participants and is unlikely to be MCAR or even MAR when conditioning only on the intervention group and stratification variables alone, all analyses will be accompanied by sensitivity analyses using multiple imputation with predictors including previously available outcome measures where possible and using chained equations when necessary. Rubin’s method for combining the results from the 25 imputations (or more if necessary such that the number of imputations exceeds the percentage of missing data) will be used. Scenario analyses will be performed to explore the robustness of findings to models of informative missingness [103]. Current or subsequent versions of Stata (14.1 or later) and R (3.3.1 or later) will be used for all analyses. Statistical analyses will be conducted by one of the co-investigators (AG) who also performed analyses on outcomes at earlier times.

## Discussion

Although interest in early life approaches to obesity prevention is high [25], much of the existing literature is based on small studies, or those with non-randomised designs [24]. In particular, relatively few large RCTs have been undertaken in the first 2 years of life. However, three recent Australian trials have demonstrated that behavioural change is challenging, but possible, at least in terms of eating behaviours and reducing sedentary time [30–32]. Only one [30] of the three [31, 32] Australian trials reported a significant impact on BMI at two years of age, an intervention benefit that was not sustained at 5 years of age [33]. However, compilation of these Australian data with our POI study in a prospective meta-analysis should help elucidate which dietary and/or activity targets, settings, or intervention approaches might be optimal for combating early excessive child growth [45].

Our POI study was novel in including a sleep intervention arm that reduced the odds of obesity at two years of age, despite no differences in sleep duration between intervention and control groups [55]. The follow-up analyses described here have been designed to determine whether variation in self-regulatory abilities in children may contribute to this intriguing observation. Our study has many strengths, including frequent high-quality assessment, good retention of participants, and use of objective measures where possible (such as 7-day accelerometry to measure sleep and physical activity). Collection of other novel measures such as of gut microbiota in this large prospective study should also allow for testing of new hypotheses thought to be important in the development of childhood obesity.

## Abbreviations

BASC-2, Behavioral Assessment System for children; BMI, body mass index; CBQ, Children’s Behavior Questionnaire; DASS 21, Depression, Anxiety and Stress Scale; DXA, dual-energy x-ray absorptiometry; FFQ, food frequency questionnaire; MAR, missing at random; MCAR, missing completely at random; NZ, New Zealand; POI, Prevention of Overweight in Infancy; RCT, randomised controlled trial

## References

[1] World Health Organization. Global strategy on diet, physical activity, and health: childhood overweight and obesity. http://www.who.int/dietphysicalactivity/childhood/en/. Accessed: 13 November 2015.

[2] Ng M, Fleming T, Robinson M, Thomson B, Graetz N, Margono C, Mullany EC, Biryukov S, Abbafati C, Abera SF (2014). Global, regional, and national prevalence of overweight and obesity in children and adults during 1980–2013: a systematic analysis for the Global Burden of Disease Study 2013. Lancet.

[3] Skinner AC, Perrin EM, Skelton JA (2016). Prevalence of obesity and severe obesity in US children, 1999–2014. Obesity.

[4] Ministry of Health. Annual update of key results 2014/15: New Zealand Health Survey. http://www.health.govt.nz/publication/annual-update-key-results-2014-15-new-zealand-health-survey. Accessed: 28 April 2016.

[5] Shibli R, Rubin L, Akons H, Shaoul R (2008). Morbidity of overweight (≥85th percentile) in the first 2 years of life. Pediatrics.

[6] Druet C, Stettler N, Sharp S, Simmons RK, Cooper C, Smith GD, Ekelund U, Levy-Marchal C, Jarvelin MR, Kuh D (2012). Prediction of childhood obesity in infancy weight gain: an individual-level meta-analysis. Paediatr Perinat Epidemiol.

[7] Gillman MW, Ludwig DS (2013). How early should obesity prevention start?. N Engl J Med.

[8] Taveras EM, Gillman MW, Kleinman KP, Rich-Edwards JW, Rifas-Shiman SL (2013). Reducing racial/ethnic disparities in childhood obesity: the role of early life risk factors. JAMA Pediatr.

[9] Malik VS, Willett WC, Hu FB (2013). Global obesity: trends, risk factors and policy implications. Nat Rev Endocrinol.

[10] Silventoinen K, Rokholm B, Kaprio J, Sørensen TI (2010). The genetic and environmental influences on childhood obesity: a systematic review of twin and adoption studies. Int J Obes.

[11] Karlsson CL, Onnerfalt J, Xu J, Molin G, Ahrné S, Thorngren-Jerneck K (2012). The microbiota of the gut in preschool children with normal and excessive body weight. Obesity (Silver Spring).

[12] Lumeng JC, Taveras EM, Birch L, Yanovski SZ (2015). Prevention of obesity in infancy and early childhood: a National Institutes of Health workshop. JAMA Pediatr.

[13] Chen X, Beydoun MA, Wang Y (2008). Is sleep duration associated with childhood obesity? A systematic review and meta-analysis. Obesity.

[14] Chan RS, Woo J (2010). Prevention of Overweight and Obesity: How Effective is the Current Public Health Approach. Int J Environ Res Public Health.

[15] Lobstein T (2004). The prevention of obesity in children. Pediatr Endocrinol Rev.

[16] West F, Sanders MR, Cleghorn GJ, Davies PS (2010). Randomised clinical trial of a family-based lifestyle intervention for childhood obesity involving parents as the exclusive agents of change. Behav Res Ther.

[17] Gerards SM, Sleddens EF, Dagnelie PC, de Vries NK, Kremers SP (2011). Interventions addressing general parenting to prevent or treat childhood obesity. Int J Pediatr Obes.

[18] Sleddens EF, Gerards SM, Thijs C, de Vries NK, Kremers SP (2011). General parenting, childhood overweight and obesity-inducing behaviors: a review. Int J Pediatr Obes.

[19] Topham GL, Page MC, Hubbs-Tait L, Rutledge JM, Kennedy TS, Shriver L, Harrist AW (2010). Maternal depression and socio-economic status moderate the parenting style/child obesity association. Public Health Nutr.

[20] Bergmeier H, Skouteris H, Horwood S, Hooley M, Richardson B (2014). Associations between child temperament, maternal feeding practices and child body mass index during the preschool years: a systematic review of the literature. Obes Rev.

[21] Wu T, Dixon WE, Dalton WT, Tudiver F, Liu X (2011). Joint effects of child temperament and maternal sensitivity on the development of childhood obesity. Matern Child Health J.

[22] Birch LL, Doub AE (2014). Learning to eat: birth to age 2. Am J Clin Nutr.

[23] Rothbart MK, Sheese BE (2011). Posner. Developing mechanisms of self-regulation in early life. Emotion Review.

[24] Blake-Lamb TL, Locks L, Perkins ME, Woo Baidal JA, Cheng ER, Taveras EM (2016). Interventions for childhood obesity in the first 1000 days. A systematic review. Am J Prev Med.

[25] Redsell SA, Edmonds B, Swift JA, Siriwardena AN, Weng S, Nathan D, Glazebrook C (2016). Systematic review of randomised controlled trials of interventions that aim to reduce the risk, either directly or indirectly, of overweight and obesity in infancy and early childhood. Matern Child Nutr.

[26] Paul IM, Savage JS, Anzman SL, Beiler JS, Marini ME, Stokes JL, Birch LL (2011). Preventing obesity during infancy: a pilot study. Obesity.

[27] Verbestel V, De Coen V, Van Winckel M, Huybrechts I, Maes L, De Bourdeaudhuij I (2014). Prevention of overweight in children younger than 2 years old: a pilot cluster-randomized controlled trial. Public Health Nutr.

[28] Mustila T, Raitanen J, Keskinen P, Saari A, Luoto R (2013). Pragmatic controlled trial to prevent childhood obesity in maternity and child health care clinics: pregnancy and infant weight outcomes (the VACOPP Study). BMC Pediatr.

[29] Navarro JI, Sigulem DM, Ferraro AA, Polanco JJ, Barros AJD (2013). The double task of preventing malnutrition and overweight: a quasi- experimental community-based trial. BMC Public Health.

[30] Wen LM, Baur LA, Simpson JM, Rissel C, Wardle K, Flood VM (2012). Effectiveness of home based early intervention on children’s BMI at age 2: randomised controlled trial. BMJ.

[31] Campbell KJ, Lioret S, McNaughton SA, Crawford DA, Salmon J, Ball K, McCallum Z, Gerner BE, Spence AC, Cameron AJ (2013). A parent-focused intervention to reduce infant obesity risk behaviors: a randomized trial. Pediatrics.

[32] Daniels LA, Mallan KM, Nicholson JM, Battistutta D, Magarey A (2013). Outcomes of an early feeding practices intervention to prevent childhood obesity. Pediatrics.

[33] Wen LM, Baur LA, Simpson JM, Xu H, Hayes AJ, Hardy LL, Williams M, Rissel C (2015). Sustainability of effects of an early childhood obesity prevention trial over time: a further 3-year follow-up of the Healthy Beginnings Trial. JAMA Pediatr.

[34] Daniels LA, Mallan KM, Nicholson JM, Thorpe K, Nambiar S, Mauch CE, Magarey A (2015). An early feeding practices intervention for obesity prevention. Pediatrics.

[35] Hnatiuk J, Ridgers ND, Salmon J, Campbell K, McCallum Z, Hesketh K (2012). Physical activity levels and patterns of 19-month-old children. Med Sci Sports Exerc.

[36] Forsyth BW, Leventhal JM, McCarthy PL (1985). Mothers’ perceptions of problems of feeding and crying behaviour. Am J Dis Child.

[37] Sepa A, Frodi A, Ludvigsson J (2004). Psychosocial correlates of parenting stress, lack of support and lack of confidence/security. Scand J Psychol.

[38] Taylor BJ, Heath A-LM, Galland BC, Gray AR, Lawrence JA, Sayers R, Dale K, Coppell KJ, Taylor RW (2011). Prevention of Overweight in Infancy (POI.nz) study: a randomised controlled trial of sleep, food and activity interventions for preventing overweight from birth. BMC Public Health.

[39] Ministry of Health, New Zealand. Well Child/Tamariki Ora. http://www.health.govt.nz/your-health/services-and-support/health-care-services/well-child-tamariki-ora. Accessed: June 2016.

[40] Cameron SL, Heath A-LM, Gray AR, Churcher B, Davies RS, Newlands A, Galland BC, Sayers RM, Lawrence JA, Taylor BJ (2015). Lactation consultant support from late pregnancy with an educational intervention at 4 months of age delays the introduction of complementary foods in a randomized controlled trial. J Nutr.

[41] Fangupo LJ, Heath A-L, Williams SM, Somerville MR, Lawrence JA, Gray AR, Taylor BJ, Mills VC, Watson EO, Galland BC (2015). Impact of an early-life intervention on the nutrition behaviors of 2-year old children: a randomized controlled trial. Am J Clin Nutr.

[42] Wen LM, Baur LA, Rissel C, Wardle K, Alperstein G, Simpson JM (2007). Early intervention of multiple home visits to prevent childhood obesity in a disadvantaged population: a home-based randomised controlled trial (Healthy Beginnings Trial). BMC Public Health.

[43] Daniels LA, Magarey A, Battistutta D, Nicholson JM, Farrell A, Davidson G, Cleghorn G (2009). The NOURISH randomised control trial: Positive feeding practices and food preferences in early childhood – a primary prevention program for childhood obesity. BMC Public Health.

[44] Campbell K, Hesketh K, Crawford D, Salmon J, Ball K, McCallum Z (2008). The Infant Feeding Activity and Nutrition Trial (INFANT) an early intervention to prevent childhood obesity: cluster-randomised controlled trial. BMC Public Health.

[45] Askie LM, Baur LA, Campbell K, Daniels L, Hesketh K, Magarey A, Mihrshahi S, Rissel C, Simes J, Taylor B (2010). The Early Prevention of Obesity in CHildren (EPOCH) Collaboration – an individual patient data prospective meta-analysis. BMC Public Health.

[46] Hart CN, Cairns A, Jelalian E (2011). Sleep and obesity in children and adolescents. Pediatr Clin N Am.

[47] Galland BC, Mitchell EA (2010). Helping children sleep. Arch Dis Child.

[48] Nielsen LS, Danielsen KV, Sørensen TIA (2011). Short sleep duration as a possible cause of obesity: critical analysis of the epidemiological evidence. Obes Rev.

[49] Carter PJ, Taylor BJ, Williams SM, Taylor RW (2011). A longitudinal analysis of sleep in relation to BMI and body fat in children: the FLAME study. BMJ.

[50] Fatima Y, Doi SAR, Mamun AA (2015). Longitudinal impact of sleep on overweight and obesity in children and adolescents: a systematic review and bias-adjusted meta-analysis. Obes Rev.

[51] Haines J, McDonald J, O’Brien A (2013). Healthy habits, happy homes: Randomized trial to improve household routines for obesity prevention among preschool-aged children. JAMA Pediatr.

[52] Wake M, Price A, Clifford S, Ukoumunne OC, Hiscock H (2011). Does an intervention that improves infant sleep also improve overweight at age 6? Follow-up of a randomised trial. Arch Dis Child.

[53] Yoong SL, Chai LK, Williams CM, Wiggers J, Finch M, Wolfenden L (2016). Systematic review and meta-analysis of interventions targeting sleep and their impact on body mass index, diet, and physical activity. Obesity.

[54] Sadeh A, Tikotzky L, Scher A (2010). Parenting and infant sleep. Sleep Med Rev.

[55] Galland B, Taylor B, Gray A, Heath A, Lawrence J, Sayers R, Cameron S, Hanna M, Dale K, Coppell K (2016). Early life prevention of obesity by targeting sleep, or food and activity: a randomized controlled trial. SLEEP.

[56] Schmid SM, Hallschmid M, Schultes B (2015). The metabolic burden of sleep loss. Lancet Diab Endocrinol.

[57] Gluckman PD, Hanson M, Zimmet P, Forrester T (2011). Losing the war against obesity: the need for a developmental perspective. Sci Transl Med.

[58] Mindell JA, Telofski LS, Wiegand B, Kurtz ES (2009). A nightly bedtime routine: impact on sleep in young children and maternal mood. Sleep.

[59] Francis LA, Susman EJ (2009). Self-regulation and rapid weight gain in children from age 3 to 12 years. Arch Pediatr Adolesc Med.

[60] Moffitt TE, Arseneault L, Belsky D, Dickson N, Hancox RJ, Harrington H, Houts R, Poulton R, Roberts BW, Ross S (2011). A gradient of childhood self-control predicts health, wealth, and public safety. Proc Natl Acad Sci U S A.

[61] Caleza C, Yañez-Vico RM, Mendoza A, Iglesias-Linares A (2015). Childhood obesity and delayed gratification behavior: a systematic review of experimental studies. J Pediatr.

[62] St James-Roberts I, Roberts M, Hovish K, Owen C (2015). Video Evidence That London Infants Can Resettle Themselves Back to Sleep After Waking in the Night, as well as Sleep for Long Periods, by 3 Months of Age. J Dev Behav Pediatr.

[63] Korkman M, Kirk UKS (2007). NEPSY – Second Edition (NEPSY-II).

[64] McClelland MM, Cameron CE (2011). Self-regulation in early childhood: Improving conceptual clarity and developing ecologically valid measures. Child Dev Perspect.

[65] Reilly JJ, Jackson DM, Montgomery C, Kelly LA, Slater C, Grant S, Paton JY (2004). Total energy expenditure and physical activity in young Scottish children: mixed longitudinal study. Lancet.

[66] Hinkley T, Salmon J, Okely AD, Crawford D, Hesketh KD (2012). Preschoolers’ physical activity, screen time, and compliance with recommendations. Med Sci Sports Exerc.

[67] Vale S, Trost S, Ruiz J, Rêgo C, Moreira P, Mota J (2013). Physical activity guidelines and preschooler’s obesity status. Int J Obes.

[68] Taylor RW, Murdoch L, Carter P, Gerrard DF, Williams SM, Taylor BJ (2009). Longitudinal study of physical activity and inactivity in preschoolers: FLAME study. Med Sci Sports Exerc.

[69] Cliff DP, Reilly JJ, Okely AD (2009). Methodological considerations in using accelerometers to assess habitual physical activity in children aged 0–5 years. J Sci Med Sport.

[70] Van Cauwenberghe E, Gubbels J, De Bourdeaudhuij I, Cardon G (2011). Feasibility and validity of accelerometer measurements to assess physical activity in toddlers. Int J Behav Nutr Phys Act.

[71] Hnatiuk J, Salmon J, Hinkley T, Okely A, Trost S (2014). A review of preschool children’s physical activity and sedentary time using objective measures. Am J Prev Med.

[72] Meredith-Jones K, Williams SM, Galland BC, Kennedy G, Taylor RW (2016). 24h accelerometry: Impact of sleep-screening methods on estimates of physical activity and sedentary time. J Sports Sci.

[73] Ly NP, Litonjua A, Gold DR, Celedon JC (2011). Gut microbiota, probiotics, and vitamin D: interrelated exposures influencing allergy, asthma, and obesity?. J Allergy Clin Immunol.

[74] Harley ITW, Karp CL (2012). Obesity and the gut microbiome: striving for causality. Mol Metab.

[75] Angelakis E, Armougom F, Million M, Raoult D (2012). The relationship between gut microbiota and weight gain in humans. Future Microbiol.

[76] Chang L, Neu J (2015). Early factors leading to later obesity: interactions of the microbiome, epigenome, and nutrition. Curr Probl Pediatr Adolesc Health Care.

[77] Kalliomaki M, Collado MC, Salminen S, Isolauri E (2008). Early difference in fecal microbiota composition in children may predict overweight. Am J Clin Nutr.

[78] Luoto R, Kalliomaki M, Laitinen K, Delzenne NM, Cani PD, Salminen S, Isolauri E (2011). Initial dietary and microbiological environments deviate in normal-weight compared to overweight children at 10 years of age. J Pediatr Gastroenterol Nutr.

[79] Scheepers LEJM, Penders J, Mbakwa CA, Thijs C, Mommers M, Arts ICW (2015). The intestinal microbiota composition and weight development in children: the KOALA Birth Cohort study. Int J Obes.

[80] Weden MM, Brownell PB, Rendall MS, Lau C, Fernandes M, Zafar N (2013). Parent-reported height and weight as sources of bias in survey estimates of childhood obesity. Am J Epidemiol.

[81] Conterno L, Fava F, Viola R, Tuohy KM (2011). Obesity and the gut microbiota: does up-regulating colonic fermentation protect against obesity and metabolic disease?. Genes Nutr.

[82] de Onis M, Onyango AW, Van den Broeck J, Chumlea WC, Martorell R (2004). Measurement and standardization protocols for anthropometry used in the construction of a new international growth reference. Food Nutr Bull.

[83] WHO Multicentre Growth Reference Study Group. WHO Child Growth Standards based on length/height, weight and age. Acta Paediatr. 2006;450(Suppl):76–85. 10.1111/j.1651-2227.2006.tb02378.x 16817681

[84] Ponitz CC, McClelland MM, Matthews JS, Morrison FJ (2009). A structured observation of behavioral self-regulation and its contribution to kindergarten outcomes. Dev Psychol.

[85] Reynolds CR, Kamphaus RW (2004). BASC-2: Behavior Assessment System for Children.

[86] Rothbart MK, Ahadi SA, Hershey KL, Fisher P (2001). Investigations of temperament at three to seven years: the Children’s Behavior Questionnaire. Child Dev.

[87] Tan CC, Holub SC (2011). Children’s Self-Regulation in Eating: Associations with Inhibitory Control and Parents’ Feeding Behavior. J Pediatr Psychol.

[88] Taylor RW, Jones IE, Williams SM, Goulding A (2000). An evaluation of waist circumference, waist-to-hip ratio and the conicity index to screen for high trunk fat, as measured by dual-energy x-ray absorptiometry in children aged 3–19 y. Am J Clin Nutr.

[89] Taylor RW, Grant AM, Williams SM, Goulding A (2009). Sex differences in regional body fat distribution from pre- to postpuberty. Obesity.

[90] Galland BC, Kennedy G, Mitchell EA, Taylor BJ (2012). Algorithms for using an activity-based accelerometer for identification of infant sleep-wake states during nap studies. Sleep Med.

[91] Galland BC, Meredith-Jones K, Gray A, Taylor BJ, Taylor RW (2016). Criteria for nap identification in infants and young children using 24-h actigraphy and agreement with parental diary. Sleep Med.

[92] Evenson KR, Catellier DJ, Gill K, Ondrak KS, McMurray RG (2008). Calibration of two objective measures of physical activity for children. J Sports Sci.

[93] Trost SG, Loprinzi PD, Moore R, Pfeiffer KA (2011). Comparison of accelerometer cut points for predicting acitivity intensity in youth. Med Sci Sports Exerc.

[94] Bruni O, Ottavianio S, Guidetti V, Romoli M, Innocenzi M, Cortesi F, Giannotti F (1996). The Sleep Disturbance Scale for Children (SDSC): Construction and validation of an instrument to evaluate sleep disturbances in childhood and adolescence. J Sleep Res.

[95] Watson EO, Heath A-LM, Taylor RW, Mills VC, Barris A, Skidmore PML (2015). Relative validity and reproducibility of a food frequency questionnaire to determine nutrient intakes of New Zealand toddlers aged 12–24 months. Public Health Nutr.

[96] Mills VC, Skidmore PML, Watson EO, Taylor RW, Heath A-LM (2015). Relative validity and reproducibility of a food frequency questionnaire for identifying the dietary patterns of New Zealand toddlers. J Acad Nutr Diet.

[97] Caporaso JG, Kuczynski J, Stombaugh J, Bittinger K, Bushman FD, Costello EK, Fierer N, Gonzalez Peña A, Goodrick JK, Gordon JI (2010). QIIME allows analysis of high-throughput community sequencing data. Nat Methods.

[98] Sauvé B, Koren G, Walsh G, Tokmakejian S, Van Uum SHM (2007). Measurement of cortisol in human hair as a biomarker of systemic exposure. Clin Invest Med.

[99] Russell E, Koren G, Rieder M, Van Uum S (2012). Hair cortisol as a biological marker of chronic stress: current status, future directions and unanswered questions. Psychoneuroendocrinology.

[100] Lovibond SH, Lovibond PF (1995). Manual for the Depression Anxiety Stress Scales.

[101] Katan BC (2013). Bias in randomised factorial trials. Stat Med.

[102] Katan BC, Morris TP (2012). Improper analysis of triala randomised using stratified blocks or minimisation. Stat Med.

[103] White IR, Horton NJ, Carpenter J, Pocock SJ (2011). Strategy for intention to treat analysis in randomised trials with missing outcome data. BMJ.

[104] Meltzer LJ, Montgomery-Downs HE, Insana SP, Walsh CM (2012). Use of actigraphy for assessment in pediatric sleep research. Sleep Med Rev.

